# Impact of a nationwide study for surveillance of maternal near-miss on the quality of care provided by participating centers: a quantitative and qualitative approach

**DOI:** 10.1186/1471-2393-14-122

**Published:** 2014-04-01

**Authors:** Adriana Gomes Luz, Maria José Duarte Osis, Meire Ribeiro, José Guilherme Cecatti, Eliana Amaral

**Affiliations:** 1 University of Campinas UNICAMP, Campinas, Brazil; 2 CEMICAMP, Campinas, Brazil; 3 Rua Alexander Fleming, 101 Cidade Universitária, 13083-881 Campinas, SP, Brazil

**Keywords:** Maternal morbidity, Near miss, Quality of care

## Abstract

**Background:**

The Brazilian Network for Surveillance of Severe Maternal Morbidity was established in 27 centers in different regions of Brazil to investigate the frequency of severe maternal morbidity (near-miss and potentially life-threatening conditions) and associated factors, and to create a collaborative network for studies on perinatal health. It also allowed interventions aimed at improving the quality of care in the participating institutions. The objective of this study was to evaluate the perception of the professionals involved regarding the effect of participating in such network on the quality of care provided to women.

**Methods:**

A mixed quantitative and qualitative study interviewed coordinators, investigators and managers from all the 27 obstetric units that had participated in the network. Following verbal informed consent, data were collected six and twelve months after the surveillance period using structured and semi-structured interviews that were conducted by telephone and recorded. A descriptive analysis for the quantitative and categorical data, and a thematic content analysis for the answers to the open questions were performed.

**Results:**

The vast majority (93%) of interviewees considered it was important to have participated in the network and 95% that their ability to identify cases of severe maternal morbidity had improved. They also considered that the study had a positive effect, leading to changes in how cases were identified, better organization/standardization of team activities, changes in routines/protocols, implementation of auditing for severe cases, dissemination of knowledge at local/regional level and a contribution to local and/or national identification of maternal morbidity. After 12 months, interviewees mentioned the need to improve prenatal care and the scientific importance of the results. Some believed that there had been little or no impact due to the poor dissemination of information and the resistance of professionals to change practice. In this second interview, a lack of systematic surveillance after the end of the study, difficulty in referring cases and changes in the leadership of the unit were mentioned.

**Conclusion:**

In the opinion of these professionals, participating in a network for the surveillance of severe maternal morbidity represented a good strategy for improving services, even in reference centers.

## Background

According to the World Health Organization (WHO) estimates, the rate of maternal mortality has fallen worldwide by 47% over the past two decades, from 400 maternal deaths per 100,000 live born infants in 1990 to 210 per 100,000 in 2010. To achieve the fifth Millennium Development Goal, effective interventions with a rapid impact would be necessary, including improving access to emergency obstetric services and qualified professionals [1]. It is believed that the great majority of deaths and complications could be avoided if appropriate healthcare were available during pregnancy, childbirth and in the postpartum. The care provided by trained professionals is recognized today as being essential in order to reduce maternal mortality and the incidence of complications resulting from pregnancy. This training refers to detect complications at an early stage and to immediately offer emergency obstetric care when necessary [2,3].

Worldwide, millions of women every year present with severe complications during pregnancy, childbirth or in the postpartum; however, the exact numbers are not yet completely known [4,5]. Therefore, maternal morbidity has been receiving more attention from investigators, particularly the most severe cases, known as maternal near-miss [6-8]. Cases of near-miss are those in which women develop life-threatening complications during pregnancy, childbirth or in the postpartum but survive by chance or because of good hospital care [2,9,10]. Recently, the WHO developed criteria for defining maternal near miss, with the objective of standardizing its identification and of being able to use this event as a sentinel for the quality of care provided to women during pregnancy, childbirth and in the postpartum [2].

The concept of improving the quality of the organizational characteristics of healthcare has been attracting more and more interest. A recent Cochrane review on the effects of audit and feedback within professional practice and the consequent results to healthcare concluded that this procedure might be effective. The impact on individuals may be slight, as with other continued medical education interventions; however, it results in a relevant impact on the work process. Its efficacy is greater when initial compliance with good practice is lower and depends on the quality of feedback supplied [11]. Another intervention with professionals, recently submitted to meta-analysis, was the strategy of continued medical education. The results were similar. The use of different interactive educational strategies, with a focus on results that are considered important by the participants, may increase impact; meetings of an educational nature, alone or as part of a strategy with other components, may be effective in changing complex behaviors [12].

These findings give further strength to the idea that healthcare professionals and services are intrinsically motivated to improve care, but may fail to realize that their practices are inadequate or out-of-date. When work processes and/or clinical practice are found to be incompatible with existing directives or best evidence, professionals may be asked to change their practices. Then the correspondent feedback must be given in an appropriate way, without attributing blame and with the focus on improving healthcare [12]. The recommendation is to establish a systematic obstetric audit and feedback in healthcare units to reduce maternal and neonatal complications [13].

A population-based study conducted in Campinas, Brazil over a three-month period aimed at standardizing the investigation and discussion of cases of severe maternal morbidity (near- miss) as a health intervention to qualify the surveillance system in cases of maternal death. The cases were identified daily at all the maternity hospitals in the city and all were discussed later with the members of the Municipal and State Maternal Death Committees to identify potential delays. The audit system implemented by the project, with participation of professionals from these committees, was instrumental in constructing the clinical reasoning, causality and predictability of cases of morbidity and mortality in accordance with the different clinical conditions, allowing identification of the levels of care that needed to be better qualified [9].

With this in mind, a group of Brazilian investigators implemented the Brazilian Network for the Surveillance of Severe Maternal Morbidity, with the participation of 27 centers in different regions of the country. The initial objective was to evaluate the frequency of severe maternal morbidity (near-miss and potentially life-threatening conditions) and the factors associated with them [14]. The activities of this network were conducted between July 2009 and June 2010. In each center, there was an investigator, who was responsible for reviewing and confirming the cases, and a coordinator, who was in charge of identifying the cases and collecting the data. Over the duration of the study, a collaborative surveillance network was running for cases of severe maternal morbidity (near-miss and potentially life-threatening conditions) that allowed the quality of care provided to these women to be evaluated [15].

It is also possible to think that the implementation of this surveillance network have produced changes in the ability of professionals to deal with cases of severe maternal morbidity, besides changes and/or reorganization of the routines in the health services. This is based on the general and well established and accepted concept that identifying earlier any problem would enable professionals to better and more appropriately manage the condition, then improving the quality of care provided. The objective of the present manuscript was mainly to present the results from the evaluation of the impact of this network based on the perceptions of the professionals involved in thestudy and on the quality of care provided to the pregnant women in the participating centers.

## Methods

A concurrently quantitative and qualitative study [16] was conducted to which all the professionals who had been involved in the Brazilian Network for Surveillance of Severe Maternal Morbidity in the 27 reference obstetric units in the various geographical regions of Brazil were invited to participate. The quantitative approach was descriptive and the qualitative approach was in the form of a summative evaluation [17].

The coordinator and the principal investigator of the project and the service manager at each obstetric unit (the director of the obstetric unit or the clinical director) were invited to participate in the study. The interviews were held at two moments: six months (stage 1) and 12 months (stage 2) after implementation of the network project. Interviewers were previously trained for this study and were experienced in conducting telephone surveys. All interviews were conducted by telephone and simultaneously recorded [18]. To guarantee anonymity, the recordings were identified only by numbers. Prior to initiating the interview, an informed consent form was read to the potential subjects and their consent, if given, was recorded.

For data collection, an instrument with two sections, a structured and a semi-structured one, was used. This instrument had been previously tested in a sample of ten professionals that were not in the final sample and some modifications were made following this evaluation. The structured section of the instrument contained two questions comprising response categories within a 7-point Likert-type scale. These questions concerned the perception of the impact of this study on the knowledge, ability or competence of the individuals interviewed to identify cases of severe maternal morbidity following its implementation. Participants were also asked questions on the relevance of the study. The semi-structured section contained questions on the participants’ perspective regarding the impact and the changes brought about by its implementation, both in relation to the professional him/herself and in relation to the obstetric unit.

In each stage of the data collection (6 and 12 months) we had 81 potential subjects: 54 local investigators and/or coordinators of the Brazilian Network for Surveillance of Severe Maternal Morbidity project, and 27 managers of the participant centers. Each potential subject was contacted by telephone at least six times before the interview was considered a loss. Even then, when telephone contact was unsuccessful, e-mail messages were sent to the individual.

In the first stage, 60 professionals were interviewed: 52 local investigators and/or coordinators (4 of these persons worked as both investigators and managers) and 8 persons who were only managers. Two researchers were not interviewed: one of the individuals in question was the principal investigator of the present study and the other was hospitalized for health problems. 15 managers could not be contacted or were unavailable due to a full agenda.

In stage 2, 62 professionals were interviewed: 48 investigators and/or coordinators (10 of these professionals had also worked as manager) and 14 persons who were only managers. One interview with an investigator was discarded because the quality of the recording did not permit analysis to be made; another two investigators were unavailable for interview during the interviewers’ working hours; and in another two cases contact proved impossible. Three managers failed to reply to the request for an interview. In addition, the principal investigator of the present study was also not interviewed at this stage.

Of the 54 interviews scheduled to be conducted with investigators and coordinators in the first stage, 52 were indeed carried out. In the other two cases, one of the individuals in question was the principal investigator of the present study and the other was in hospital. Eight managers were also investigators/coordinators and 11 other managers could not be contacted or were unavailable due to a full agenda.

In stage 2, 48 of the 54 interviews planned with investigators and coordinators were completed. One interview with an investigator was discarded because the quality of the recording did not permit analysis to be made; another two investigators were unavailable for interview during the interviewers’ working hours; and in another two cases contact proved impossible. Of the 27 managers scheduled to be interviewed, 10 were also investigators or coordinators and three possible subjects failed to reply to the request for an interview. The higher success rate in obtaining interviews in stage 2 was partly due to the fact that telephone contact had been made previously with the clinical directors of each service.

The NVIVO® software program, version 9.0 was used to codify the interviews, organize and analyze the qualitative data. A thematic content analysis was performed [17]. The interviews carried out in stage 1 were transcribed in their totality. In stage 2, the interviews were listened to in their entirety but only the segments referring to new themes were transcribed. The themes discussed by the individuals interviewed were identified and categories of analysis were defined. These categories were defined based on the study objectives, on the guide used to conduct the interviews, and also on the material that emerged from the participants speech during the interviews: 1.Reasons given by interviewees for justifying their opinion of a positive outcome: change in how cases are identified, Better organization in the center/routine/standardization of staff activities, Dissemination of knowledge at local/regional level/changes in health policies, Proposed changes in routines/protocols, Review of severe cases, Reduction in maternal morbidity and mortality, Local and/or national diagnosis of maternal morbidity.2. Reasons given by interviewees for justifying their opinion of a negative outcome: Poor dissemination of information, resistance of the professionals to changes in protocols, lack of surveillance after the end of the network, difficulty in referring cases after morbidity is identified and difficulties with leadership transition. This list of categories was used to compile a coding manual, which was then used to codify the transcripts and analyze the interviews conducted in both stages of the study. This process was performed by 2 co-authors (MJDO and MR) and discussed and reviewed by the 2 others co-authors (AL and EMA). Quotations from the transcripts were used to illustrate the results presented. The interviews conducted in stage 1 were identified by the letter “A” and those conducted in stage 2 by the letter “B”.

The research project was approved by the internal review board of the School of Medical Sciences, University of Campinas (UNICAMP) under protocol number 1067/2010.

In this paper, the qualitative results are presented according to the perception of the professionals interviewed regarding the impact of implementing the network on the quality of services provided by their respective centers.

## Results

A total of 122 interviews were conducted, 60 in stage 1 and 62 in stage 2. Of the 68 individuals who participated in the study in the first and/or second stage, 50% were women and 66% were over 40 years of age. The majority (86.7%) of the interviewees were physicians (Table 1). Of the 27 participating centers, five were secondary level institutes and 22 were tertiary level, with four municipal hospitals, nine state hospitals, 10 federal hospitals and four private hospitals. With respect to localization, 12 were situated in the north, northeast and mid-west of the country, and 15 were in the south and southeast (data not presented as table).

**Table 1:** Characteristic of the professionals interviewed during the first and second stages of the study

**First stage**			
**Characteristics**	**Coordinators**	**Investigators**	**Managers**
***Sex***			
Female	17	11	2
Male	10	14	6
***Age (years)***			
25 a 39	16	5	1
40 or older	11	20	7
***Profession***			
Physician	20	25	8
Nurse	7	0	0
***Total number of individuals***	27	25	8
**Second stage**			
**Characteristics**	**Coordinators**	**Investigators**	**Managers**
***Sex***			
Female	15	11	4
Male	10	12	10
***Age (years)***			
25 a 39	15	4	1
40 or older	10	19	13
***Profession***			
Physician	19	23	14
Nurse	6	0	0
***Total number of individuals***	25	23	14

### Quantitative data

The great majority of the coordinators/investigators interviewed considered that the importance of implementing the network was considered above average or very high, according to a Likert-type scale used, in relation to their previous individual knowledge and experience with severe maternal morbidity and in comparison with other peer professionals (93.1%). The same occurred with 90% of the managers. With respect to their knowledge, ability or competence to identify cases of severe maternal morbidity, 95% of the coordinators/investigators considered that having participated in the network had made them competent or expert on the subject. The same was reported by 90% of the participating managers (data not presented as tables).

### Qualitative data

#### Positive impact

In the majority of the interviews held at stage 1 (6 months), the professionals understood that implementation of the network had produced positive effects and this was confirmed at 12 months, as shown in Table 2. The categories that were only identified in the interviews held in stage 2 (12 months) were the need to improve prenatal care and the importance of the findings at a scientific level.

**Table 2:** Why interviewees believe that the surveillance project on severe maternal morbidity had a positive feedback

**Reasons given by interviewees for justifying their opinion of a positive outcome**	**After 6 months**	**After 12 months**
Change in how cases are identified	χ	χ
Better organization in the center/routine/standardization of staff activities	χ	χ
Dissemination of knowledge at local/regional level/changes in health policies	χ	χ
Proposed changes in routines/protocols	χ	χ
Review of severe cases	χ	χ
Reduction in maternal morbidity and mortality	χ	χ
Local and/or national diagnosis of maternal morbidity	χ	-
Improvements required in prenatal care	-	χ
Importance at scientific level	-	χ

In the evaluation of the majority of the participants, the network project contributed towards introducing changes to improve the service. According to the interviewees, there was an improvement in the knowledge of the health professionals in relation to severe maternal morbidity. It was mentioned that participating in this project helped clarify the criteria established by the WHO, resulting in a better understanding by the participants.

*I believe that this work helped … with the concepts, the concepts became clearer, I was able to fix the concepts of the criteria of severe maternal morbidity better in my mind and exactly because I was more aware of these concepts, these criteria, it is easier for me to identify these cases today, but it was because of this, basically because of this”.* (Case 34A, coordinator).

Thirty-three of the subjects stated that improvements occurred in the organization of the center and in its routine, with the team activities being standardized. Several individuals at different moments mentioned that a standardization in attitudes enabled cases to be identified more easily. In particular, this achievement was a consequence of the standardization of the team’s clinical activities, including those of duty staff, and of defining data that should be mandatorily collected, but that had previously received little attention.

In addition, this standardization contributed towards changes in protocols. Seven of the interviewees stated that the network encouraged a review to be made of severe cases as a means of identifying where and how problems occurred. Individuals stated that reviewing cases allowed feedback to be given to colleagues with respect to care that was possibly inadequate or delays that may have occurred in any action.

*“I believe it was worthwhile, because, as I said, it is about reevaluating each case, right, discussing it with the chief and finding out why some cases, despite having the criteria established for severe maternal morbidity, are not followed-up as they should be…so then we reevaluate them.* (Case 43A, coordinator).

In both stages of the project, some of the interviewees mentioned that the main impact of the project was in disseminating knowledge, not only within their own center but also in other spheres (at local and regional level).

*“Yes, because from the time it was implemented…all this training, particularly the…of the head of the ICU, right? Dr. X, we also began to…disseminate this knowledge within our own institute and at regional events”.* (Case 62A, manager).

At 12 months, the most obvious improvements in the center, such as ensuring compliance with protocols, was evident even to physicians who had not been present during the implementation phase of the Brazilian Network for the Surveillance of Severe Maternal Morbidity. Some stated that strict compliance with protocols leads to excellent long-term results. Others mentioned that the greatest contribution to the health of these pregnant women was the reduction in the rates of maternal mortality and morbidity. In one interview, an individual stated that there had been a reduction in the mortality rate at the center.

*“Definitely. After the network was implemented, we had no more maternal deaths. In 2009, at the beginning, before the network, we had four”.* (Case 23A, investigator).

It was only in the interviews held at stage 2 that the importance of the study in “scientific” terms was mentioned, i.e. the results of implementing the network were published in journal articles that ratified the importance of the network itself.

*Again I would say yes…nevertheless, I believe that it adds to current scientific knowledge on the subject; conduct is now professional. [So it would be scientific conduct. What do you mean by scientific conduct?] The analyses are now complete, a short time ago Dr. Y sent us two very interesting papers to analyze…with very interesting statistical and epidemiological methodology that confirmed what we already knew in the clinic, but showing the results scientifically, confirming the importance of the criteria”.* (Case 49B, investigator).

There were reports in the interviews of proposed changes in the center after the network was implemented, with the objective of solving problems based on identified shortcomings. There was also mention of proposals to improve the center either by constructing an ICU or other facilities that would allow them to provide better care.

*“I believe that (by talking about?) the subject, ensuring that…perhaps by making the center recognize its failings, seeing what is wrong within the center and also what is wrong with the center within the health system and where these problems are, trying to solve some of these problems, from the simplest to the most complex, so I think this experience should be expanded to all maternity hospitals, managing the problems to ensure that they are really prevented (in terms of severe conditions?) and solving these problems”.* (Case 20B, coordinator).

The interviewees mentioned the importance of the project and the need to create health policies based on the results found.

*“It’s like I already said. I think that if the results are shown to the managers, the health secretary or the managers of the unit itself, this is going to result in changes in behavior, in routines, protocols will be implemented”.* (Case 40B, investigator).

Some interviewees pointed out a need to improve prenatal care and spoke of its importance in improving the health of the pregnant woman.

*“In the sense of alerting doctors and the population itself to the importance of prenatal care. Its importance in preventing these diseases, alerting the government to invest more in hiring more doctors, invest more in basic healthcare, in the primary healthcare network to promote prevention and ensure that quality prenatal care is provided”.* (Case 2B, manager).

### No effect or little effect

The opinions of interviewees that implementation of the network had had no effect at all or little effect gave rise to certain categories of analysis that were identified at six months and were still present at twelve months: poor dissemination of the information and colleagues’ resistance to changes in protocols. Nevertheless, some categories appeared only at twelve months such as the lack of surveillance following the conclusion of the network project, the difficulty encountered in referring cases after they were identified, and difficulties due to a change in the unit’s leadership (Table 3).

**Table 3:** Why interviewees believe that the surveillance project on severe maternal morbidity had a little/no impact

**Reasons given by interviewees for justifying their opinion of a negative outcome**	**After 6 months**	**After 12 months**
Poor dissemination of information	χ	χ
Resistance of the professionals to changes in protocols	**-**	χ
Lack of surveillance after the end of the network	**-**	χ
Difficulty in referring cases after morbidity is identified	**-**	χ
Difficulties with leadership transition	**-**	χ

At stage 1, around one-third of the interviewees believed that no changes had occurred following implementation of the surveillance network. The motives given were: the WHO criteria were already known; information remained limited to those involved in the project and was not disseminated to the entire team; a possible wrong choice of project coordinator; and even failings within the institute.

Four of the individuals interviewed considered that including the formal criteria was interesting, but that it had not resulted in any great effect. They mentioned a poor dissemination of information and proposed changes in the team of professionals.

*“Yes…nothing much changed. Yes, it was already established [routine at the center] and…yes…as a result of the project, we put together a flowchart to investigate…But, no, this flowchart is no longer in use”.* (Case 55A, investigator).

Some said that changes had yet to take place in the routine of the center, since they were waiting to see the results of the study conducted on the implementation of the Brazilian Network for the Surveillance of Severe Maternal Morbidity.

*For now, the identification of cases, that part of the network, has resulted in very little change in the routine at this center. The idea is to implement changes from now on based on the results that were obtained”.* (Case 29A, investigator).

It was also reported that, although the WHO criteria were important for identifying severe cases, it was very difficult to refer or treat these cases.

*“Exactly because of what I said before. I think that, it’s…even with these conditions that the …the World Health Organization is…establishes, we are able to evaluate this patient and, identify that she was very seriously ill. What are missing are the necessary conditions to allow us to follow up this patient, to treat this patient or refer her for treatment. [Would you like to add anything else?] Yes, I would. I am coordinator of this institute but I am not here every day. So, my view is quite superficial, based on what has been expressed in the interviews. Perhaps if I worked here every day, maybe I would have a different opinion. But, of the cases that I saw and followed up, from what I saw, from the study, that’s my opinion”.* (Case 20B, investigator).

In stage 2, some interviewees spoke of physicians’ cultural resistance to changing the way in which they manage cases in order to comply with protocols, of the deep-rooted habits in the institute, a feature that was not reported in stage 1. Another interesting aspect that emerged from the interviews with the managers in this respect refers to a disassociation between the university (research) and the center (care).

*“There are some cultural obstacles, not all the doctors try to follow the protocols, ‘this is my way of doing it’, they do not want to follow or comply with a protocol. From one work shift to another, there is no continuity in the criteria adopted, in those habits, to classify a patient as more or less severe, needing more or less attention”.* (Case 16B, investigator).

*“…and I see that, as a manager, although this is a teaching hospital, there is a distance between the provision of care and teaching, and this is a basic factor, it’s…from understanding the need to disseminate this information and the criteria for the identification of maternal morbidity…I believe that greater commitment from the faculty is crucial and also commitment from the preceptors, who are often concerned only with providing care”.* (Case 9B, manager).

One interviewee mentioned that there had been no changes in the routine at the center and that this was because of the change in the unit’s leadership.

“Nothing has changed yet because there was a change in leadership and, it’s…it’s still…the new boss is still thinking about how to do things differently, these routines…”

It was also mentioned that there was no continuity with respect to an active search for cases of near-miss after the project had finished. One person stated that this was because there were few very serious cases in that institute.

*“Unfortunately, we did not manage to implement it after the end of the network project, to keep actively searching for cases of near-miss. However, since we work in a place where the risk is low, we really have very few cases of near-miss over a year, so, really, a broader surveillance system is not justified because we really have very few cases, right, so there are these two mitigating factors in this respect”.* (Case 26B, investigator).

## Discussion

This study showed that, in the view of the healthcare professionals involved in a project to implement surveillance in cases of severe maternal morbidity (the Brazilian Network for the Surveillance of Severe Maternal Morbidity initiative), positive changes occurred in the majority of the participating centers and, indirectly, apparently also in the quality of care provided to the women. In other words, increased awareness made it easier to identify women at risk of severe morbidity and also increased the center’s ability to review cases. Ensuring that more professionals were aware of the criteria resulted in more effective medical management of these cases. This finding is similar to the results encountered in another study on obstetric audit [19,20].

Although the quantitative analysis showed that the vast majority of the professionals believed that the effect of the network project on them in particular was slight because they already worked with high-risk pregnancies, they pointed out many positive aspects. These were related to changes in the ways cases were identified; improvements in the organization of the center, its routine procedures and the standardization of team activities; dissemination of information at local and regional levels; changes in health policies; proposed changes in routines and protocols; and review of severe cases.

The results of having participated in this project, with access to new information and new technology, the adoption of new practices and changes within centers, are in agreement with the observations of other authors [11,12]. Integration of infrastructure, human and financial resources, information, technology and activities aimed at improving quality may permit a center to direct its resources towards patients’ needs, particularly with respect to prevention and care, but also towards the needs of the professionals insofar as the work environment is concerned [21]. Actually, difficulties in integration between infrastructure, human and financial resources were also mentioned by some of the participants.

This evaluation led us to perceive that reflecting on the positive and negative points and on the situation of maternal morbidity and mortality in this country was important in promoting changes in the attitudes and behavior of the investigators themselves and of their colleagues. It was also found that during the surveillance of cases for the project, a significant change took place in the care offered, with a potential reduction in maternal morbidity and mortality. However, changes in the structure of the services and in the protocols instituted appear to be accepted more easily after the final results are presented.

The changes at individual level or in the centers were perhaps those that would have had the greatest impact if a real audit were made; however, this project did not incorporate all the steps involved in audit and feedback. In fact, the project consisted of developing a complex system for checking data, and also the quality of the care, by identifying delays in providing the care [22]. However, audit was not performed on an individual basis with the centers or the professionals and feedback was not given to them, as it would be in a true audit.

According to the WHO, audit and feedback form a cycle involving the following steps: (i) establishing criteria for better practice; (ii) observing how current practice contrasts with standard practice, e.g. with a local audit panel; (iii) providing feedback on conclusions and establishing local standards (often done by the same panel); (iv) implementing changes; and (v) evaluating the results, returning then once again to step (i) [11,13]. To increase the effect of local audit, these steps and the perception of the healthcare professionals regarding the local audit process require a lot more work before they can be put into practice.

Several studies have shown that the efficacy of audit depends on how feedback is given to the professionals involved. The PRECEDE model (Predisposing, Reinforcing, Enabling Constructs in Educational Diagnosis and Evaluation), proposed by Green and Kreuter, shows that various elements such as guidelines, educational lectures and conferences may predispose individuals to behavioral changes; however audit and feedback have been shown to be very useful in solidifying behavioral changes [23].

The nature of learning organizations, or as organizations of knowledge, has been described as a cyclic process in which individuals need to recognize, interpret and adopt measures to manage tasks and potential safety risks in their daily work. Argyris and Scho theorized that learning through errors and failures involves describing both the situation in which the individual identifies an error and corrects it as well as the situation in which the institute learns from the error and changes the conditions that have contributed towards underlying errors. This latter manner of learning includes taking measures to eliminate recurrent problems, thus contributing to the organization’s ability to improve performance [24].

It was not within the objectives of the network project to complete all the steps in the audit and feedback process, but the intention was to fulfill all the stages of the project itself; however, because of funding constraints the opportunity for the centers to discuss the proposed changes was lost. This missed opportunity to be able to review the results and the effects of having participated in the project was mentioned by some individuals and is understood to represent an essential step, which may now prove simpler in view of the greater availability of resources such as video conference systems and other similar tools.

These results show the relevance of the proposal of a training program to establish a public audit policy to collaborate with healthcare professionals and centers in improving care provided to pregnant women, with the objective of reducing the incidence of severe maternal morbidity and mortality in Brazil.

These results confirm that participating in a research project involving the surveillance of a sentinel health event is a good strategy for improving services, even those provided by reference centers. Nevertheless, optimizing the results from this intervention in the centers depends on completing the audit-feedback loop, with proposals for improvement followed by subsequent reevaluation. Some subsequent effects or a more in-depth self-criticism only seems to appear after a certain interval of time (in this case 12 months), meaning that these interventions need to be reviewed after longer periods of time to enable actions to be proposed to resolve problems that were only recognized at a later stage.

## Conclusion

In the opinion of these professionals, participating in a network for the surveillance of severe maternal morbidity represented a good strategy for improving services, even in reference centers.

## Competing interests

The authors declare that they have no competing interests.

## Authors’ contributions

The idea for the study arose in a group discussion among AGL, EA and JGC. The first version of the manuscript was drafted by AGL and EA, and then complemented with the suggestions of the others. AGL and MR were responsible for interviews. MR and MJMDO were responsible to define categories of analysis. All authors contributed to the development of the study protocol and approved the final version of the manuscript.

## Pre-publication history

The pre-publication history for this paper can be accessed here:

http://www.biomedcentral.com/1471-2393/14/122/prepub
